# Evaluation of Low Volume Roads Surfaced with 100% RAP Millings

**DOI:** 10.3390/ma15217462

**Published:** 2022-10-25

**Authors:** Adam J. T. Hand, Prathapan Ragavan, Nicole G. Elias, Elie Y. Hajj, Peter E. Sebaaly

**Affiliations:** Department of Civil and Environmental Engineering, University of Nevada Reno, Reno, NV 89557, USA

**Keywords:** asphalt concrete, low volume roads, reclaimed asphalt pavement, pavement design, cold milling

## Abstract

The sustainability of roadway construction has rapidly been gaining attention within the pavement industry. The pavements examined in this study are in a Northern Nevada county with many of the roadways categorized as low volume roads. The county began surfacing rural roads with 100% Reclaimed Asphalt Pavement (RAP) millings, without any design considerations for decades. These pavements have provided satisfactory performance with little to no maintenance for their intended purpose for 25–30 years. The presented research revealed RAP milling surfaced roads with layer coefficients between 0.18 and 0.30, and design thicknesses ranging from 5 to 11 inches.

## 1. Introduction

**Figure 1:**
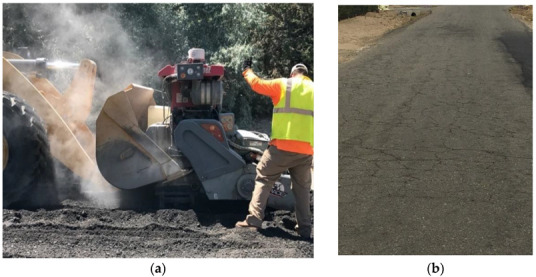
(**a**) Asphalt reclaimer on front end loader doing full depth reclamation, (**b**) Existing field cracking of RAP milling surfaced roads.

The infrastructure system of lightly populated counties in the United States (U.S.) is mainly founded on Low Volume Roads (LVR). These LVR are used on a daily basis by residents and businesses to commute and vitalize the economic services of many rural areas. The roadway system evaluated in this study belongs to a county located in the northwestern part of Nevada. Many of the existing LVR were built over 25 years ago by simply spreading and compacting Reclaimed Asphalt Pavement (RAP) millings, then applying an emulsion fog seal one month after placement. Interestingly, these 100% recycled pavements have provided satisfactory performance for their intended purpose for over two decades with little to no maintenance as per Figure 1. As the county population grew, a limited number of these LVR transitioned to collector streets and were subsequently surfaced either with a chip seal or a thin Asphalt Concrete (AC) layer. Others remained in service with no real maintenance until recently [1]. These pavements were more cost effective than gravel roads, which require frequent annual maintenance, and they offered other sustainable benefits including no dust being generated from vehicles which is a common complaint with gravel roads. This technique consisting of 100% RAP without additional virgin material has not been commonly adopted by local agencies in the United States due to lack of a well-defined design, construction, and maintenance guidelines. Several research studies have emphasized the advantages associated with the use of RAP in asphalt pavements, including significant savings in cost and energy along with the environmental benefits due to reduction in the disposal of waste during maintenance and rehabilitation activities [2,3]. The results of life-cycle cost analysis (LCCA) and life-cycle assessment (LCA) conducted by Aurangzeb and AL-Qadi in 2014, showed savings in costs and energy use, along with reduction in greenhouse gas emissions associated with higher RAP doses in asphalt mixtures [4]. The incorporation of RAP in asphalt mixtures has been evaluated by many international studies, including Reyez-Ortiz et al. who evaluated the impact of partial and total replacement of aggregates by RAP on the mechanical response of dense graded asphalt mixtures in Columbia, with 3% neat asphalt binder. The results suggest that mixtures with 100% RAP obtained the maximum indirect tensile strength and resilient modulus under dry and wet conditions [5]. In 2013, Zaumanis et al. evaluated the effect of several rejuvenators on asphalt mixtures with high RAP doses varying from 40 to 100%. This study determined the indirect tensile strength and creep compliance for rejuvenated 100% RAP mixtures, to assess the embrittlement of the mixture at −10 °C. Consequently, four of the nine tested rejuvenators reduced RAP mixture susceptibility to low temperature embrittlement by reducing the extracted binder consistency to the required level [6]. Yi et al. investigated the feasibility of using epoxy asphalt with 100% RAP mixtures in China, by comparing their relative performance to virgin epoxy asphalt mixtures. The 100% RAP mixtures with epoxy showed improved rutting performance, relative to virgin mixtures, with similar low temperature resistance and moisture susceptibility between both mixture types. Despite a lower fatigue resistance on the mix, the authors highlighted the potential to recycle 100% RAP mixtures with the addition of epoxy asphalt [7].

Despite the wide incorporation of RAP in asphalt mixtures based on numerous previous studies in the literature, few research studies have evaluated 100% RAP mixtures without the addition of any virgin material (e.g., asphalt binder, rejuvenator, asphalt epoxy). Noureldin and Abdelrahman analyzed the use of 100% RAP materials as base course layer, while determining an appropriate M_R_ model to simulate the RAP performance under the impact of different parameters (i.e., water content, dry density, freeze–thaw cycles). Based on the measured and predicted M_R_, the three models selected by the authors to best simulate RAP base course were the Mechanistic Empirical Pavement Design Guide (MEPDG), Pezo, and Witzack models [8]. Song and Ooi concluded that the resilient modulus of 100% RAP mixtures was higher than virgin aggregates and presented a model to predict the M_R_ of RAP, Hawaiian basaltic virgin aggregates, and their blends. The model included the effect of RAP dose, stress level, water content, and density, while indicating good estimation for the Hawaiian aggregate data set [9]. In 2018, Plati and Cliatt suggested similar modulus results between 100% RAP material and virgin aggregates base layer, mostly at increased confining pressures. Moreover, the level of confining stress had the main influence on the RAP resilient modulus, with a reduced impact from the deviator stress [10].

Figure 1 shows an asphalt reclaimer mounted on a loader that is used by the county for patching operations and can be used to in-place recycle 100% RAP millings from existing roads. Many of the RAP milling surfaced roads have far exceeded their intended design life, and some sections need rehabilitation while some are still intact with significant block cracking but are still performing well, as shown in Figure 1. A survey done by the Environmental Protection Agency, the Federal Highway Administration, and the National Asphalt Pavement Association (NAPA), that was reported to Congress indicated that asphalt pavement is America’s Number 1 recycled product [11]. Additionally, the report indicated that the use of RAP has increased at a much higher rate than any other material, where more than 89.2 million tons of RAP were employed during the 2019 construction season in pavement reconstruction and rehabilitation activities. The county is interested in recycling the RAP milling surfaced roadways again, in place, as well as constructing more using a large county owned RAP millings stockpile, based on structural pavement design and updated construction guidelines. The county did not have a rehabilitation technique documented for existing RAP milling surfaced roads and wanted a structural design method along with new construction guidelines.

**Figure 2:**
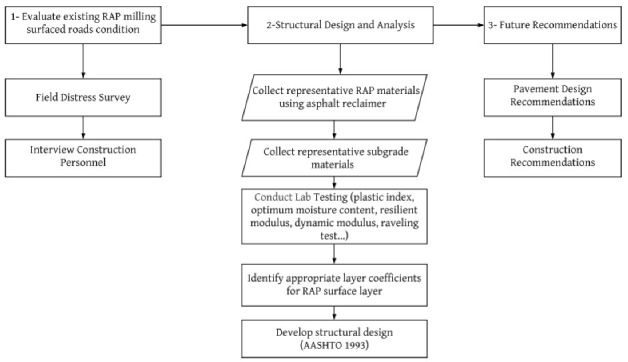
General experimental plan.

The objective of this research study was initiated by evaluating the current status of the RAP milling roadways built 25 plus years ago, through field distress survey and by interviewing the construction personnel on construction practices followed when building these roadways. Afterward, the design guidelines for construction and rehabilitation of RAP milling surfaced roads in the county, were developed consistent with the 1993 American Association of State Highway and Transportation Officials (AASHTO) Guide for Design of Pavement Structures [12]. Accordingly, the corresponding findings and recommendations were made for further construction practices and rehabilitation of the RAP milling surfaced roadways again. To accomplish this sequence of objectives, the experimental plan outlined in Figure 2 was followed: 

## 2. Project Evaluation, Construction and Maintenance

### 2.1. Project Evaluation and Construction Procedure

The RAP milling surfaced roads were originally constructed by placing 8 inches of RAP millings, adding water, blending and grading, and compacting without any binding agent. The pavements were then opened to traffic for approximately 30 days to allow kneading of the surface, after which an application of emulsion was sprayed on the surface at rates between 0.08 to 0.13 gallons per square yard. The existing millings surfaced roads were inspected to assess performance and identify typical existing pavement distresses. County personnel involved in construction of the roads were also interviewed about construction practices used for the original construction [1]. 

The predominant pavement condition included what appeared to be high severity and extent block cracking and fatigue cracking, although most of the pavements were still intact due to the low traffic volumes. A few sections had minor potholes or skin patching and some edge deterioration existed where there was no shoulder backing. Many of the surveyed roadways were still intact in acceptable condition, while others required enough patching that rehabilitation was recommended, thus suggesting the need for construction and rehabilitation guidelines [1]. Overall, the LVR were still providing a reasonable level of service for their intended purpose, in some instances even after 30 years of service. It should be mentioned that one LVR milling project became a major collector over time, hence a portion of it was surfaced with 2 inches of Hot Mix Asphalt (HMA), while surfacing the other portion with a double chip seal application. This project was still performing 11 years after the chip seal application over a geotextile. A county employee that worked on the construction of the roads was interviewed and indicated that it was believed that constructing RAP milling surfaced roads early in the construction season, so they were exposed to traffic during the warm summer months was a good practice. The following steps describe the original construction procedure used 25 to 30 years ago, for building RAP milling surfaced roads:

1. RAP millings with 100% passing the 2 inches sieve and 85–100% passing the 1 inch sieve material was placed on the subgrade (no preparation), then mixed while adding the required amount of water for compaction.
2. RAP millings were graded adequately 8 inches thickness along with appropriate cross slope.
3. The RAP millings material was compacted with steel drum rollers to refusal density.
4. Shoulder backing was placed or pulled up and compacted.
5. The roadway was open to traffic for 30 days.
6. After 30 days a fog seal with Cationic Slow Set (CSS)—1 h emulsion at an application rate of 0.08 to 0.13 gallons/yd^2^ of undiluted emulsion is applied.

### 2.2. Maintenance 

Maintenance of RAP milling surfaced roads was minimal and limited to localized patching.

## 3. Laboratory Testing and Analysis

**Figure 3:**
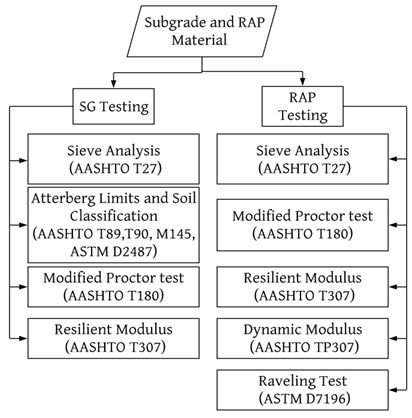
Summary of laboratory testing for SG and RAP.

This research evaluated different types of RAP and subgrade materials from the county including: two representative subgrade sources identified herein as SG1, SG2 and two different RAP sources denoted herein as RAP1 and RAP2. One collected from a RAP stockpile located by County Airport and the other from a reclaimer sampled from a pre-overlay repair activity Reno, Nevada. The flowchart in Figure 3 summarizes the laboratory testing performed on the subgrade and RAP materials, along with the corresponding standard procedures.

### 3.1. Soil Testing

The subgrade materials were classified using the particle size analysis and Atterberg limits following three methods per the United States Department of Agriculture (USDA) soil web survey, AASHTO, and American Society for Testing and Materials (ASTM) ([13,14,15]). Optimum Moisture Content (OMC) and maximum dry density were determined based on developed moisture-density relationships. The moisture-density curves were used to prepare resilient modulus test samples, which were further employed for structural design and analysis.

#### 3.1.1. Sieve Analysis

**Figure 4:**
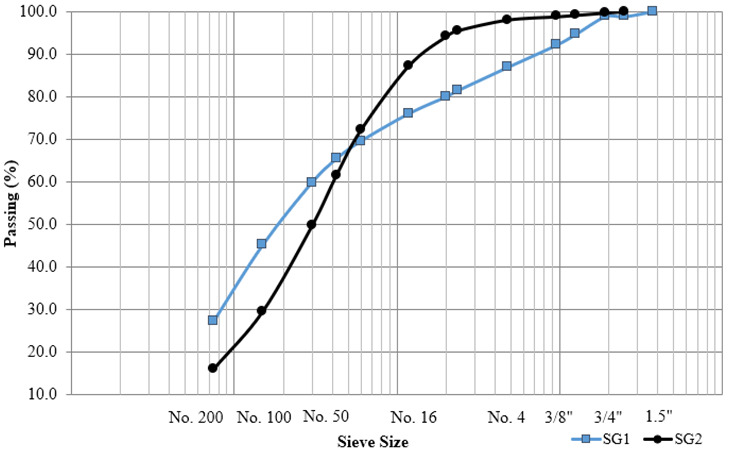
Subgrade soil gradations.

Sieve analyses were performed in accordance with AASHTO T27 Standard Method of Test for Sieve Analysis of Fine and Coarse Aggregates [16]. Figure 4 represents the 0.45 power chart comparing the two subgrade materials and shows that the SG1 material was finer than the SG2.

#### 3.1.2. Atterberg Limits

The Atterberg Limits of the subgrade soils comprise the liquid limit, plastic limit, and plastic index values, performed in accordance with AASHTO T89 Standard Method of Test for Determining the Liquid Limit of Soils, and AASHTO T90 Standard Method of Test for Determining the Plastic Limit and Plasticity Index of Soils ([17,18]). The subgrade Atterberg limits are shown in Table 1. The SG1 exhibited plastic behavior while SG2 was non-plastic. 

The soil classification of the subgrade materials was determined per AASHTO M-145 Standard Specification for Classification of Soils and Soil-Aggregate Mixtures for Highway Construction Purposes [14], American Society for Testing and Materials (ASTM) D-2487 Standard Practice for Classification of Soils for Engineering Purposes (Unified Soil Classification System) methods [15], and the United States Department of Agriculture (USDA) soil classification system [13]. The SG1 was classified as A-2-4, SM, and A-2 per AASHTO, ASTM, and USDA, respectively. Whereas SG2 was classified as A-2-4, SM, and A-1 per AASHTO, ASTM, and USDA, respectively.

#### 3.1.3. Moisture Density Relationships

**Figure 5:**
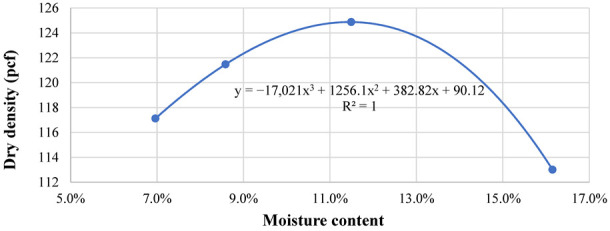
Moisture density curve for SG1 material.

This laboratory test employed to obtain the maximum dry density and the OMC of the evaluated soils, was according to AASHTO T-180 Standard Method of Test for Moisture-Density Relations of Soils Using a 4.54 kg (10 lb) Rammer and a 457 mm (18 inches) Drop [19]. The OMC and the maximum dry density summarized in Table 2, were determined using the relationship between the soil density and moisture content. An example of the moisture-density relationship developed for SG1 material is illustrated in Figure 5.

#### 3.1.4. Resilient Modulus Testing

**Figure 6:**
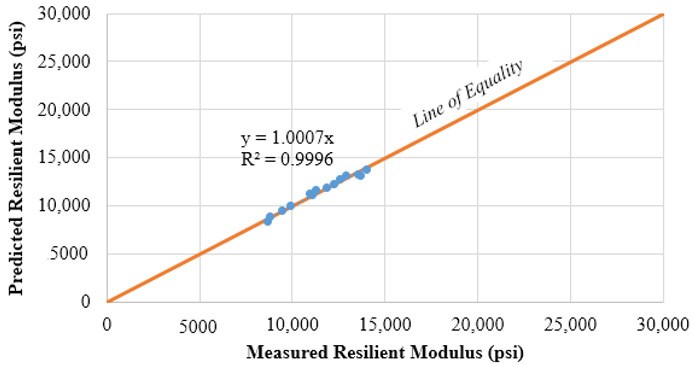
Uzan model for SG2 material.

The resilient modulus (M_R_) constitutes a fundamental property of unbound materials in a pavement structure, which is defined as the ratio between the applied axial deviator stress and recoverable axial strain. M_R_ can be obtained by subjecting cylindrical specimens to a cyclic axial load using a triaxial test. The M_R_ testing was performed per AASHTO T-307 Standard Method of Test for Determining the Resilient Modulus of Soils and Aggregate Materials [20]. The subgrade materials were compacted at the OMC using a vibratory hammer to a target maximum dry density of 90 %. Thereafter, the cyclic axial load with a loading time of 0.1 s followed by a rest period of 0.9 s, was applied in 16 sequences with different axial cyclic stresses and static confining stresses predefined in the testing machine control software as prescribed in AASHTO T-307. Consequently, the M_R_ results were employed to develop non-linear models for each subgrade material. The Uzan model presented in Equation (1) was selected, thus a regression analysis was carried out for best fit coefficient determination (R^2^) [21].

(1) $$M_{R}={K\theta }^{n}\sigma_{d}^{m}$$

where:

M_R_ = Resilient Modulus (psi);
K, n, m = Regression coefficients;
θ^n^ = Bulk Stress (psi);
σ_d_ = Deviator Stress (psi);

The regression coefficients of the Uzan model for both subgrade materials are summarized in Table 3, revealing a good correlation as indicated by the R^2^ in Figure 6. For pavement design input, the subgrade M_R_ values were determined at a depth of 6 inches below the interface of the 8 inches RAP milling surface and the subgrade. The 3D-Move analysis software was used in an iterative process, where a subgrade modulus was assumed and the modulus under a standard single axle single tire load was computed using 3D-Move [21]. This process was repeated until reaching a satisfactory convergence level. The SG1 resilient modulus was equivalent to 17,000 psi and 19,000 psi for RAP milling surface layer Air Voids (AV) of 15% and 22%, respectively. Fifteen percent was selected as the typical air voids level of well compacted Cold In place Recycling (CIR), which is mainly composed of RAP millings. The theoretical maximum dry density determined from the moisture-density relationship, was equivalent to 22% AV. It is worth mentioning, that 22% AV coincided with field density of some CIR sections in Nevada, observed to perform poorly. Whereas the SG2 resilient modulus was 8000 psi and 9000 psi for RAP millings surface layer AV of 15% and 22%, respectively.

### 3.2. Reclaimed Asphalt Pavement (RAP) Materials

The following section summarizes the laboratory testing conducted on RAP millings material with the aim of determining a representative layer coefficient. It is commonly known that RAP millings contain aged asphalt binder, thus may partially act as a partially bound material in the compacted state. However, RAP milling behavior cannot be categorized as the same bound material of hot mix asphalt, typically used for flexible roadway surfaces. Therefore, RAP millings can also be considered as unbound material, since no binding agent is added during the material preparation and construction process. As a consequence, the laboratory testing performed on RAP millings evaluated the materials as bound in terms of dynamic modulus testing and unbound through the resilient modulus test.

#### 3.2.1. Sieve Analysis

**Figure 7:**
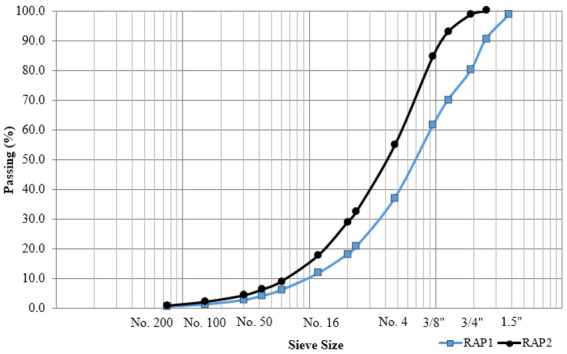
Gradation plots for both RAP sources.

The particle size analysis of the RAP milling was performed per AASHTO T27 Standard Method of Test for Sieve Analysis of Fine and Coarse Aggregates [16]. As per Figure 7, The RAP2 millings from the reclaimer came out to be finer than RAP1 stockpile material. The reclaimer milling had 100% passing the 1 inch sieve, while the RAP1 stockpile material had 100% passing the 2 inches sieve.

#### 3.2.2. Moisture Density Relationship

The moisture density relationships were developed for the RAP milling materials treating them as unbound material, per AASHTO T-180 Standard Method of Test for Moisture-Density Relations of Soils Using a 4.54 kg (10 lb) Rammer and a 457 mm (18 inches) Drop [19]. The moisture density relationships for both RAP milling sources were developed, and the OMC are summarized in Table 4 along with their maximum dry density.

#### 3.2.3. Resilient Modulus Testing

**Figure 8:**
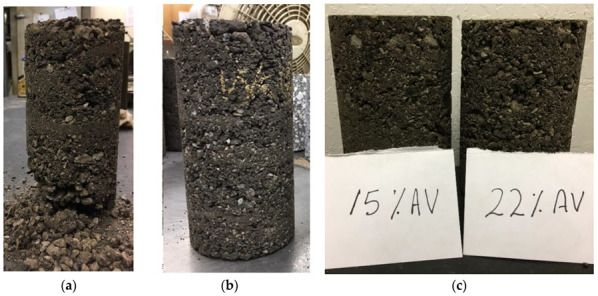
RAP milling resilient modulus test specimens before (**a**) and after (**b**,**c**) modification to AASHTO T-307 sample preparation.

**Figure 9:**
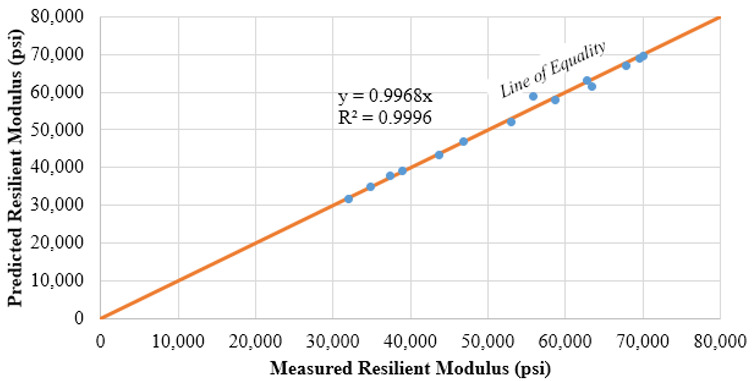
Universal model for RAP1 stockpile material.

The resilient modulus test was performed in accordance with AASHTO T-307 Standard Method of Test for Determining the Resilient Modulus of Soils and Aggregate Materials [20]. The RAP millings were considered as unbound material, mixed with water at the OMC, then conditioned overnight. Unfortunately, the samples collapsed upon extrusion from the mold as shown in Figure 8, and the specimen preparation of this test had to be adjusted in order to avoid sample collapse. Accordingly, the compacted samples were conditioned prior to extrusion from the mold for 48 h at 60 °C. This modification was done with the aim of maintaining adequate specimen integrity for the resilient modulus test, while noting that this experiment was executed without any moisture due to the 60 °C used during specimen preparation. The test outcome was evaluated using the non-linear regression analysis for the best fit of the Universal model shown in Equation (2) [21]. The regression coefficients of the Universal model for both RAP materials are summarized in Table 5, while providing an adequate fit as shown in the example of Figure 9. The range of measured/predicted M_R_ between 32,000 and 70,000 psi, per Figure 9, is lower than the range found by Plati and Cliatt between 6000 and 35,000 psi for 100% RAP materials [10].

(2) Mr=k1paθpak2τoctpa+1k3

where:

k_1_, k_2_, k_3_ = Regression coefficients;
p_a_ = Atmospheric pressure (psi);
θ = Bulk stress (psi);
τ_oct_ = Octahedral shear stress (psi);

#### 3.2.4. Dynamic Modulus Testing

**Figure 10:**
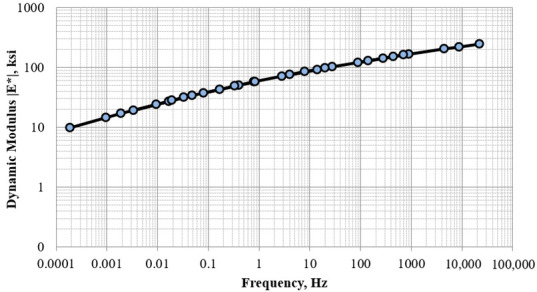
Master curve for RAP2 milling materials at 22% AV and 20 °C reference temperature.

The dynamic modulus test was conducted in accordance with AASHTO TP-79 Standard Method of Test for Determining the Dynamic Modulus and Flow Number for Asphalt Mixtures Using the Asphalt Mixture Performance Tester (AMPT) [22]. The specimens were prepared using a Superpave gyratory compactor with a 4 inches diameter mold. This was done so the specimens could be directly tested in the AMPT without coring or cutting, which would have led to sample damage. This experiment was carried out on specimens compacted at two different AV levels of 15 and 22%. The dynamic modulus test was conducted at 4 °C, and 20 °C with frequencies of 0.1, 1 and 10 Hz. At the high temperature of 40 °C, the test frequencies adopted were 0.01, 0.1, 1 and 10 Hz. The results were used to develop the master curve for each RAP millings source and air void combination, as shown in Figure 10. The dynamic modulus value is a fundamental property, which is a critical input for pavement designs per the 1993 AASHTO Guide for Design of Pavement Structures, as it ultimately defines the layer coefficient for the surface layer [12]. According to the climate data, 21.1 °C (70 °F) was selected as the pavement temperature and 10 Hz was selected as the loading frequency for subsequent pavement designs.

#### 3.2.5. Raveling Test

Based on the identification of some raveling during the field inspection, raveling was given consideration for RAP milling surfaced roads, especially early in the pavement life. Raveling tests were conducted similarly to cold mixes, based on ASTM D7196 Standard Test Method for Raveling Test of Cold Mixed Emulsified Asphalt Samples [23], with a target AV of 22%. Table 6 summarizes the raveling test results showing 2 to 3% mass loss, while noting that the maximum allowable mass loss criteria for cold mix used by most road agencies is 2%. Although the mass loss slightly exceeded 2% for most RAP samples, the specimens did not contain any binder and raveling was not a commonly observed distress in the county pavements after 20 years of service.

## 4. Structural Design

The structural pavement design for LVR was carried out following the 1993 AASHTO Guide for Design of Pavement Structures, using the PaveXpress software (Version 3.0) [12,24], while recognizing some limitations behind applying the assumptions of this software for such particular material. The design parameters were obtained from historical records of the corresponding county. Furthermore, a sensitivity analysis was performed to determine the impact of selected reliability level and different soil types on the required design thickness. According to the County Design Criteria and Improvement Standards, 20 years analysis period is commonly used for these LVR [25], while assuming an initial serviceability index P_o_ of 4.2 for low traffic speed. Terminal serviceability values of 1.5 and 2.0 were adopted due to the very low traffic volume and reduced truck percentages on these roadways. The county classifies these LVR for light vehicles and garbage trucks, hence specifying 10,000 or 50,000 Equivalent Single Axle Load (ESALs) as load levels along with 4% traffic growth factor, and 0.5 directional distribution [25]. Moreover, reliability levels of 50% and 75% were analyzed, which represent the extreme values relative to this roadway classification.

The subgrade layer properties were obtained from laboratory testing performed on the two types of representative soils under the anticipated stress state, while considering the seasonal adjustment, per the Manual for Designing Flexible Pavements in Nevada Using AASHTOW are Pavement-ME Design [26]. As previously mentioned, the RAP milling materials were evaluated as unbound (M_R_) and bound (E*) layers, which resulted in different modulus values for the same layer. According to the 1993 AASHTO Guide for Design of Pavement Structures [12], the layer coefficients in Table 7 were determined corresponding to the representative resilient or dynamic modulus values measured on both RAP millings.

**Figure 11:**
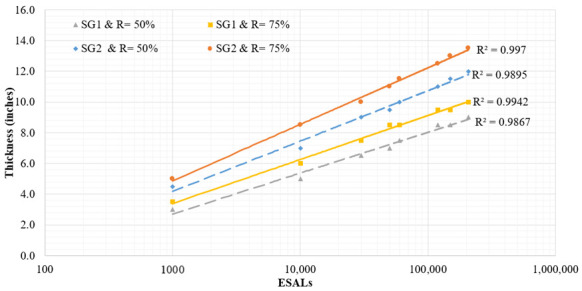
RAP milling surface layer thickness based on E* value for 22%AV (a_1_ = 0.18).

The LVR pavement designs were developed using PaveXpress, which is a free cloud-based pavement design software, founded on the 1993 AASHTO Guide for Design of Pavement Structures [12]. Several plots were developed for the design thickness versus ESALs, for different combinations of design inputs as shown in the example in Figure 11, illustrating the sensitivity of required RAP millings thickness to subgrade stiffness and reliability level as a function of traffic level. When the RAP milling material was treated as an unbound layer, the recommended design thickness determined based on the region, traffic level and the quality of roadbed soil is summarized in Table 8. Additionally, the structural design at 78% and 85% relative in place density for RAP millings, when considered as bound material, is presented in Table 9.

## 5. Conclusions and Recommendations

The county has successfully built LVR surfaced with 100% RAP millings at low cost, which required minimal maintenance, while providing good service for up to 30 years. However, the thickness of the surface layer has always been 8 inches based on engineering judgment. Based on the limited materials available in the County the pavement designs were recommend per the layer coefficients with the 78% density to be conservative. Following to the field inspections, materials testing, and pavement design presented in this study, the following conclusions were drawn:

1. RAP milling surface for LVR can be effectively designed per the 1993 AASHTO Guide for 10,000 or 50,000 ESALs, while considering several types of subgrade soils with different resilient modulus.
2. RAP millings resulted in similar AASHTO layer coefficient estimates per the AASHTO T-307 M_R_ test results and the AASHTO TP-79 E* test results at 22% AV.
3. For construction practices, the asphalt reclaimer was practically used to in-place recycle 100% RAP millings from the existing roads.
4. When treated as unbound materials, the sample conditioning and preparation techniques of RAP millings in the laboratory had to be adjusted to fabricate specimens with the required integrity for testing under these conditions.
5. The range of M_R_ found in this study for 100% RAP materials varied between 32,000 and 70,000 psi, which is lower than 6000–35,000 psi found by other research studies.
6. The stiffness (modulus) of RAP millings surface exhibited high sensitivity to density, hence RAP millings should be compacted to the refusal density during construction.
7. The structural design outcome suggests that for low traffic (10,000 ESALs), the surface layer thickness could be constructed as low as 5.5 inches even at 78% in place density.
8. RAP millings surfaced layer thickness is sensitive to the design reliability level, hence an increase from 50% to 75%, increased the required RAP milling thickness up to 1 inch.
9. Both RAP milling sources from the reclaimer and the stockpile were similar, with the reclaimer RAP being slightly finer.

It is worth noting that limited work has been done in this area and that sample preparation for resilient modulus and dynamic modulus testing had to be modified from the standard test methods in order to fabricate 100% RAP milling samples for testing, which required some conditioning in the mold at 60 °C. This was the most successful method identified in the research, though it may not be representative of freshly compacted RAP millings. As such this may have some influence on the test results observed. Additionally, specimens were prepared at relatively low densities. Following to the laboratory testing results and pavement design analysis, the following recommendations were made for adequate recycling and reconstruction of these RAP milling surfaced roads:

1. Based on their cost effectiveness and relative performance, the county should continue using the RAP milling surface for low volume roads.
2. The top 6 inches of subgrade shall be scarified and compacted to at least 95% relative compaction prior to the RAP millings layer.
3. As per the pavement design analysis, and past performance of 8 inches thick RAP milling surfaced roads, the county should consider using a 6 inches thick RAP millings surface thickness for the low traffic category (<10,000 ESALs) roads and 8 inches for the high traffic category (10,000 to 50,000 ESALs) roads.
4. Consider doing future RAP milling surfaced road in place rehabilitations, both partial and full depth using the asphalt reclaimer, while adding chip seals or some other wearing surface treatments at intersections to prevent raveling if necessary.
5. Construct RAP milling surfaced roads early in the construction season, so they get exposed to traffic during the warm summer months prior to fog sealing and onset of the winter.
6. A RAP millings gradation specification should be implemented with 100% passing the 2 inches sieve and 85–100% passing the 1 inch sieve and allow for the use of supplemental fines to improve compactability of the millings.
7. Require that RAP millings be compacted to refusal density as their stiffness is sensitive to in place density.
8. Determine the density of existing RAP milling surfaced roads to serve as a basis for implementing a compaction specification.
9. For partial or full depth rehabilitation of RAP milling surfaced roads, the surface layer could be pulverized in place with a reclaimer, while following the same construction practices described above.

According to the particular practices considered in the pavement design and construction of RAP milling surfaced roads, future research is recommended to consider:

1. The fines contents of the RAP used is low, which could result in difficulty with compaction in the field and thus low in-place density. In scenarios like this, adding supplemental fines and adjusting gradation specifications can be helpful.
2. It is worth noting that limited work has been done in this area and that sample preparation for resilient modulus and dynamic modulus testing had to be modified from the standard test methods in order to fabricate 100% RAP milling samples for testing, which required some conditioning in the mold at 60 °C. So, additional sample preparation techniques should be evaluated.
3. Account for RAP millings seasonal variation in the pavement design, noting that AASHTO 1993 design guide considers the seasonal variation solely in the subgrade.
4. The design analysis and findings presented above refer to the particular RAP sources investigated in this paper. Moreover, this laboratory evaluation process shall be performed for any new RAP source considered in the future.
5. As well, it is worth including several RAP sources in further studies for a universal construction and expand the analysis procedure. The evaluation of a wider range of RAP sources can depict the impact of the RAP binder content and stiffness.
6. Develop laboratory performance models for future mechanistic analysis for 100% RAP materials.

## Figures and Tables

**Table 1:** Plasticity index of subgrades.

Property	SG1	SG2
Liquid Limit	24.9	-
Plastic Limit	21.4	-
Plasticity Index	3.5	NP

NP: Non-Plastic.

**Table 2:** Maximum dry density and optimum moisture content of subgrades.

Property	SG1	SG2
Maximum dry density (pcf)	123.9	125.1
Corrected moisture content (%)	10.0	8.9

**Table 3:** Regression coefficients of Uzan model for subgrade materials.

	SG1	SG2
K	9007.959	3623.333
n	0.257	0.454
m	0.029	−0.071

**Table 4:** RAP millings maximum dry density and optimum moisture contents.

Property	RAP1	RAP2
Maximum dry density (pcf)	116.3	112.6
Corrected moisture content (%)	7.4	8.0

**Table 5:** Regression coefficients of Universal model for RAP materials.

Model Coefficients	RAP1 Millings	RAP2 Stockpile
k_1_	2382.303	1263.488
k_2_	0.380	0.000
k_3_	0.090	1.771

**Table 6:** Summary of raveling test results.

	RAP1 Millings		RAP2 Stockpile	
	S1	S2	S1	S2
Initial Mass (g)	2598.3	2652.1	2641.4	2734.5
Final Mass (g)	2525.3	2581.1	2581.5	2682.5
Mass Loss (%)	2.8	2.7	2.3	1.9
Average (%)	2.7		2.1	

**Table 7:** Summary of modulus and layer coefficients.

Source	Resilient Modulus (psi)	Dynamic Modulus (psi)	
		22% AV	15% AV
RAP1 Stockpile	52,392	100,500	205,200
RAP2 Millings	42,779	75,300	201,700
Average	47,586	87,900	203,450
Layer Coefficient	0.19	0.18	0.30

**Table 8:** Recommended RAP millings surface layer thickness if considered unbound.

Quality of Roadbed Soil	Traffic Level	Thickness (Inches)
Very good (>8200 psi)	Low (10,000 to 30,000)	4.0
	Medium 30,000 to 60,000)	7.0
	High (60,000 to 100,000)	9.0

**Table 9:** RAP millings surface layer thickness.

Subgrade Classification	Minimum in Place Density	RAP Millings Surface Layer Thickness (Inches)			
		75% Reliability		50% Reliability	
		10,000 ESALS	50,000 ESALs	10,000 ESALS	50,000 ESALs
SG1	78%	6.0	8.5	5.0	7.0
SG2	78%	8.5	11.0	7.0	9.5
SG1	85%	4.0	5.0	3.5	4.5
SG2	85%	5.0	7.0	4.5	6.0
